# Grades, Student Satisfaction and Retention in Online and Face-to-Face Introductory Psychology Units: A Test of Equivalency Theory

**DOI:** 10.3389/fpsyg.2016.00673

**Published:** 2016-05-10

**Authors:** David Garratt-Reed, Lynne D. Roberts, Brody Heritage

**Affiliations:** ^1^School of Psychology and Speech Pathology, Curtin UniversityBentley, WA, Australia; ^2^School of Psychology and Exercise Science, Murdoch UniversityMurdoch, WA, Australia

**Keywords:** equivalency theory, online learning, introductory psychology, group-work, student retention

## Abstract

There has been a recent rapid growth in the number of psychology courses offered online through institutions of higher education. The American Psychological Association has highlighted the importance of ensuring the effectiveness of online psychology courses (Halonen et al., 2013). Despite this, there have been inconsistent findings regarding student grades, satisfaction, and retention in online psychology units. Equivalency Theory (Simonson, 1999; Simonson et al., 1999) posits that online and classroom-based learners will attain equivalent learning outcomes when equivalent learning experiences are provided. We present a study of an online introductory psychology unit designed to provide equivalent learning experiences to the pre-existing face-to-face version of the unit. Using quasi-experimental methods, academic performance, student feedback, and retention data from 866 Australian undergraduate psychology students were examined to assess whether the online unit developed to provide equivalent learning experiences produced comparable outcomes to the ‘traditional’ unit delivered face-to-face. Student grades did not significantly differ between modes of delivery, except for a group-work based assessment where online students performed more poorly. Student satisfaction was generally high in both modes of the unit, with group-work the key source of dissatisfaction in the online unit. The results provide partial support for Equivalency Theory. The group-work based assessment did not provide an equivalent learning experience for students in the online unit highlighting the need for further research to determine effective methods of engaging students in online group activities. Consistent with previous research, retention rates were significantly lower in the online unit, indicating the need to develop effective strategies to increase online retention rates. While this study demonstrates successes in presenting students with an equivalent learning experience, we recommend that future research investigate means of successfully facilitating collaborative group-work assessment, and to explore contributing factors to actual student retention in online units beyond that of non-equivalent learning experiences.

## Introduction

In contemporary higher education there is a significant movement toward offering online units and courses as an alternative to traditional, face-to-face study (e.g., Mandernach et al., 2012; Xin et al., 2015). This applies to the discipline of psychology, despite faculty skepticism regarding teaching psychology online (Tanner et al., 2009; Mandernach et al., 2012). Indeed, the American Psychological Association (APA) has indicated the importance of ensuring the effectiveness of online psychology units (Halonen et al., 2013). Where a unit is offered in face-to-face and online format, it is important to demonstrate that online students are not at a disadvantage to their classroom-based peers (or vice-versa). Consequently, it is pertinent to examine the equivalence of learning outcomes for students who study psychology online compared to those studying in the face-to-face mode. When comparing the effectiveness of online units with face-to-face units, three variables are of importance. The first of these is student grades, the second is student satisfaction, and the third is student retention (Bernard et al., 2004b; Lyke and Frank, 2012; Xin et al., 2015).

Research on student grades in online psychology units has, to date, produced inconsistent findings. For example, Xin et al. (2015) found that, despite no pre-existing differences in grade point average, students in a traditional undergraduate exercise psychology course performed better on the examination and on overall course grade when compared to students in the online or hybrid versions of the course. Similar results were obtained by Edmonds (2006) upon analyzing the examination results of 175 undergraduates who completed a general psychology unit either online or in face-to-face mode between 1998 and 2003. Students in the face-to-face classroom performed significantly better on examinations after controlling for prior academic performance (there were no differences before applying this control). In contrast, Lyke and Frank (2012) found no differences in the performance on unit quizzes of online compared to face-to-face students in a theories of counseling course. Other studies have mirrored this finding of no difference in student grades (e.g., Lawson, 2000; Graham, 2001; Waschull, 2001; Dell et al., 2010) between the modes of unit delivery. However other research has indicated that students studying online psychology courses achieve higher grades than those in the equivalent classroom-based course (e.g., Maki et al., 2000; Taylor, 2002; Upton and Cooper, 2003; Nguyen, 2013). However, it has been suggested that students who are driven to study online are likely to be more organized and self-motivated than their classroom-based peers (Lapsley et al., 2008), highlighting the need for studies that randomly assign students to study mode (something that is not always feasible or ethically defensible). Poirier and Feldman (2004) randomly allocated students to either an online or face-to-face method of teaching in an introductory psychology course. They found that online students (*n* = 12*)* outperformed face-to-face students (*n* = 9) on exams, but that there were no differences in assignment grades between the two groups. However, given the small sample size in Poirier and Feldman’s study, these results cannot be generalized with confidence. To date, there is no definitive answer as to whether online psychology units foster similar student grades to face-to-face versions of those units.

The inconsistent findings regarding student grades in psychology-specific units and courses are mirrored in the literature comparing online and face-to-face classes across disciplines (e.g., Wang and Newlin, 2000; Poirier and Feldman, 2004). The variability in study results is reflected in meta-analytic effect sizes indicating no overall difference in student grade outcomes for online and face-to-face units, despite widespread variability in individual study findings (e.g., Bernard et al., 2004a,b; Sitzmann et al., 2006; Lahti et al., 2014). It seems that the question of whether online units (psychology-specific or otherwise) *can* produce educational outcomes comparable to face-to-face courses has been settled (Bernard et al., 2004b; Borokhovski et al., 2012). However, the question of whether and when they actually *do* appears to be more complicated, as indicated by the inconsistency in results of studies comparing student grades across the two delivery media. Bernard et al. (2004b) suggested that that a variety of factors, including instructional techniques, can lead to students in online units achieving different grades to their classroom-based peers, contributing to this inconsistency in findings throughout the literature.

One reason for the inconsistency in student grades is that, in most cases, online units are designed with the assumption that online learning is fundamentally different to face-to-face learning, in that learners and instructors are separate from one another. As a result, the majority of online courses have not been designed to provide directly equivalent learning experiences to their face-to-face counterparts (Karatas and Simsek, 2009). Consequently, most studies comparing face-to-face and online learning do so using courses that have not been specifically designed to be equivalent, even if the instructor, content, and/or some other variables are the same (Karatas and Simsek, 2009). Consequently, it is difficult to ascertain whether grade differences observed are the result of variables related to study mode, or instead reflective of differences in course structure and content presentation (e.g., Clark, 1994).

Equivalency Theory (Simonson, 1999, p. 7; Simonson et al., 1999) is based on the notion that “instructional experiences are essential to learning” and that no student, regardless of study mode, should be forced to endure lesser instructional experiences. Consequently, students who study in the online mode require learning experiences that are specifically tailored to their learning environment. This theory stipulates that online and classroom-based learners will attain equivalent learning outcomes only when equivalent learning experiences are afforded to them and that, therefore, online units should be designed to provide equivalent learning experiences to face-to-face units: “Such an approach suggests that course designers create learning experiences of equivalent value for learners regardless of the course delivery medium, allowing that the experiences themselves could be different” (Lapsley et al., 2008, p. 2). This assertion is consistent with Clark (1994) argument that content delivery, rather than format, is likely to be the key variable determining student outcomes. It is also consistent with meta-analytic findings from Sitzmann et al. (2006) that online and face-to-face units produced equivalent student outcomes when the same instructional methods (such as providing online lectures for online students, rather than written materials) were used. Consistent with this, Bernard et al. (2004b, p. 108) suggested that their meta-analytic findings indicated that online courses should be made “more like face-to-face instruction” to be maximally effective. Dell et al. (2010) suggested that class format is less important than the educational strategies employed in teaching psychology classes.

Nevertheless, few studies have set out to directly test Equivalency Theory by comparing student grades across delivery modes whereby the online unit has been designed to be directly equivalent to its face-to-face counterpart. It is therefore pertinent to compare student learning outcomes in online units and face-to-face units where the instructional methods (including provision of feedback) have been designed to be equivalent (Lapsley et al., 2008). This should provide a more stringent test of whether online and face-to-face units afford students similar learning opportunities. It should also facilitate further exploration of other factors that might mediate differences in outcomes. In the current study this is achieved through the comparison of an online and face-to-face version of an introductory psychology unit that have been designed to be equivalent.

Beyond the consideration of student grades, student satisfaction is important because it positively predicts student retention and is linked to student learning outcomes (Lyke and Frank, 2012). Mixed results have been reported when comparing student satisfaction in face-to-face and online psychology courses. Poirier and Feldman (2004) reported that students enrolled in an online introduction to psychology course were more satisfied with their instructors and with the amount and quality of interactions than were their classroom-based counterparts, although there were no differences in overall course satisfaction. However, Taylor (2002) found that introductory psychology students were more satisfied with the face-to-face course than its online equivalent, despite overall high levels of satisfaction. Maki et al. (2000) also found that online introductory psychology students were less satisfied with the unit than were their face-to-face counterparts, despite achieving higher grades. Waschull (2001) reported no difference in course satisfaction in two studies comparing online and face-to-face students in an introductory psychology course.

Meta-analytic studies across disciplines provide further insight into student satisfaction across media. In their meta-analysis, Sitzmann et al. (2006) reported that levels of student satisfaction were similar for online and classroom-based courses. Allen et al. (2002) conducted a meta-analysis which suggested that online students prefer online learning formats involving more visual information, including visualization of the instructor (compared to text-only). Boling et al. (2012) found similar results in a more recent qualitative study.

Again, it is important to examine student satisfaction with online learning in a psychology unit designed to provide equivalent learning experiences to the face-to-face version of the unit. It remains unclear whether equivalent learning experiences will result in equivalent levels of satisfaction for students, as indicated by studies showing that online students can achieve higher marks, yet be less satisfied with the unit, compared to face-to-face students (e.g., Maki et al., 2000).

Another important factor to consider, in terms of the effectiveness of online units, is student retention. Online courses typically involve much higher attrition rates than face-to-face courses (El-Tigi and Branch, 1997; Olson and Wisher, 2002; Van Doorn and Van Doorn, 2014), possibly due to feelings of isolation caused by lack of face-to-face interaction. From a review of the literature, Bowers and Kumar (2015) suggested that higher rates of attrition that are typically observed in online courses are related to students’ low perceived sense of connectedness and perceived lack of instructor presence. Consequently, “carefully designed interactions, faculty student contact and ongoing instructor feedback” (Bowers and Kumar, 2015, p. 29) are critical for student retention. There has been limited research regarding student attrition/retention in online psychology courses, although evidence suggests higher attrition rates compared to face-to-face classes (Neff and Donaldson, 2013). Nevertheless, Nguyen (2013) did not find different rates of retention for face-to-face and online psychology courses across 92 different psychology classes within the same institution.

The current study therefore aims to compare student grades and satisfaction, as well as retention rates, in online and face-to-face versions of an introductory psychology unit. Unlike previous studies in this area, the online unit was explicitly designed with Equivalency Theory (Simonson, 1999) in mind, with the aim of providing equivalent learning experiences as those afforded in the face-to-face version of the unit.

The online version of introductory psychology was designed to be equivalent to the face-to-face version that was running concurrently. We provide the following summary of the design of the face-to-face and fully online psychology units to provide context to the forthcoming analyses.

In terms of the format of presentation to students, both versions of the unit were run through a learning management system (Blackboard^TM^), with relevant course materials posted into online folders for student access. The unit coordinators (DG-R and BH) posted announcements relating to course materials and assessments which were equivalent for both study modes. Discussion Boards within Blackboard were used for posting and answering assessment and general unit-related questions in both versions of the unit. The same textbook was used in both versions of the unit.

Content organization in the online version of the unit was divided into the same six modules covered in the face-to-face version of the unit. Each module focused on different topic areas in psychology (e.g., personality) and a new module was available for students every two teaching weeks, corresponding to the timing of the equivalent module release schedule in the face-to-face unit. This was done to enhance equivalence between the study modes by allowing the unit coordinators to provide focused feedback on each individual module for online students at the same time as providing that feedback for the face-to-face students. To start each module, online students clicked on the module heading in Blackboard. Module content was presented in sequenced pages, allowing students to progress through the module content in a sequential order that reflected the content ordering in the face-to-face version of the unit.

In terms of lecture delivery, lectures in the face-to-face unit consisted of 50 min presentations, which were recorded and could be re-watched by students at their convenience. Lecture content for the online students consisted of brief (typically 5-15 min) lecture segments, specifically recorded for the online unit, which provided the same content as lectures in the face-to-face unit, but in smaller chunks (e.g., Kahn, 2012, as cited in Glance et al., 2013). Consequently, online students were not required to conduct extra reading compared to face-to-face students, unlike in many online units (e.g., O’Neill and Sai, 2014). This design decision aimed to enhance equivalence between the study modes. Lecture segments were typically presented by the same staff member who presented the face-to-face lectures. In addition, following each lecture segment, linking text invited students to participate in various activities that were designed to be equivalent to tutorials in the face-to-face unit. For example, they would be invited to use the Blog posts to discuss and apply the content of lecture segments or invited to complete questionnaires online and discuss the outcomes in the same manner as students would in the face-to-face tutorials. Blogs in particular were designed to provide interaction opportunities between students that would occur in tutorial classes. The equivalency-based design of the online unit, including the visual presentation of material (Allen et al., 2002), and the provision of equivalent support and guidance and opportunities for interaction between students (a known sources of dissatisfaction with online learning, e.g., Kahu et al., 2013), was therefore intended to maximize the opportunities for student achievement, satisfaction, and retention.

In an attempt to ensure equivalence, assessments in both delivery modes of the unit were designed to be identical where possible. Assessments in both versions of the unit consisted of a written assignment, a multiple-choice electronic test, a group presentation assignment, and a multiple-choice examination. The written assignment, electronic test, and examination were identical for online and face-to-face students. All assignments were submitted online through the learning management system, ensuring an equivalent experience for students in both versions of the unit. Students in the online unit who lived in the Perth metropolitan region attended campus to sit the electronic test and examination, whereas those outside of this area sat invigilated assessments involving the same questions. A challenge in attempting to design an equivalent group presentation assessment experience between delivery modes was noted by the unit coordinators. Students in the face-to-face unit were required to present a 15-min group presentation to an audience of their peers relating to a topic in the unit. Online students were required to do a similar activity, whereby they worked in groups to prepare a presentation. However, to ensure that students were not disadvantaged due to lack of access to appropriate technology, students were required to submit a PowerPoint presentation, with speaker notes. Although this was designed to approximate equivalence to the classroom-based presentations, it is unclear to what extent this assignment was actually providing equivalent learning experiences for students, especially given Biasutti’s (2011) finding that online students typically are dissatisfied with the requirement to conduct group work. A summary of the differences between the face-to-face and online unit is presented in **Table 1**.

**Table 1:** A comparison of the online and face-to-face versions of an introductory psychology unit.

	Face-to-face version	Online version
Content Release Schedule	Weekly	Fortnightly
Lesson Content Presentation	PowerPoint slides presented in class, whiteboard illustrations, paper handouts	Tailored video lecture and written content, arranged modularly by topic, interactive quiz/revision activities via online quiz software
Lecture Availability	In-person (lecture theater) and iLecture recordings post-lecture	Short video lecture segments (∼5–15 min) on sections of a module topic, specifically recorded for the online version
Note-taking/reflective tools	Handwritten/typed notes in class	Private journal within Blackboard (link provided during module when asking reflective questions), public discussion boards
Assessments	Written Assignment, electronic test (taken on-campus), Group Presentation Assignment (collaborated on in-person), End of semester examination (taken on-campus)	Written Assignment, electronic test (taken on-campus, or mailed to student if outside metro area), Group Presentation Assignment (collaborated on via private discussion boards on Blackboard), End of semester examination (taken on-campus, or mailed to student if outside metro area)
Peer interaction	Discussion with in-class peers and tutor	Public and private discussion boards, blog post discussion topics (one per module)

### Aims

We propose that a comparison of student grades, satisfaction, and retention in the two versions of this unit will provide a test of Equivalency Theory. In addition, this research should provide further data in terms of the effectiveness of teaching psychology units online. Through evaluation of student comments, it will additionally provide insight into which aspects of the equivalent online unit were perceived as effective, and which aspects may benefit from further refinement in order to provide equivalent learning experiences. The examination of student satisfaction ratings by delivery modes will also provide useful information as to whether online psychology units, designed to provide equivalent learning experiences to their face-to-face counterparts, can foster a sense of satisfaction with the learning process. This is a particularly important question in light of current trends toward increasing the number of online courses available at institutions of higher education (e.g., Xin et al., 2015). In this context, an understanding of the factors that promote student satisfaction, and consequent retention (Lyke and Frank, 2012) is critical.

This study will use quasi-experimental methodology to compare retention rates, student grades, and student satisfaction across face-to-face and online study delivery modes of an introductory psychology 1st-year undergraduate unit. Given the exploratory nature of the research, no specific hypotheses are proposed. The quantitative analysis is supplemented by qualitative analysis of student evaluation feedback in response to open-ended questions regarding aspects of the unit that they enjoyed, and aspects that they believed needed improvement.

## Materials and Methods

### Participants

Existing administrative data for an introductory psychology unit at an Australian university from Semester 1, 2013 was used in this research. In total 866 undergraduate university students (810 face-to-face students, 56 online students) were enrolled in this unit. Students self-selected into either the face-to-face or online version of the unit. The data were de-identified before use in this study, and demographic data such as age and gender were not available. An a-priori power analysis indicated a minimum sample size of 34 participants in the online student group, and 482 face-to-face students, was required to have sufficient power (0.80) to detect a medium *(d* = 0.50) effect in the group mean difference analyses. The available sample size therefore exceeded this minimum requirement.

### Measures

#### Final Unit Mark

For each student the final mark for the unit was calculated by summing weighted marks across all assessments in the unit, with a possible maximum value of 100.

#### Specific Assessment Marks

For each student, raw scores for each of the four assessments completed in the unit were available: Written assignment (out of 20), electronic test (out of 30), group presentation (out of 30), and final examination mark (out of 30).

#### Student Satisfaction

Student evaluation data for the unit was available as part of the university’s student feedback process (eVALUate; see Oliver et al., 2008). Students were invited to provide both quantitative (rating of items) and qualitative (comments) feedback toward the end of the study period. Students rated their satisfaction with each of 11 items on a four-point Likert-style scale, ranging from *Strongly Agree* to *Strongly Disagree*, with *Unable to Judge* provided as an additional response option. An example item is “I make best use of the learning experiences in this unit”. Two open ended items asked “Please comment on the most helpful aspects of [unit name]” and “Please comment on how you think [unit name] might be improved”. The de-identified aggregated feedback from students was made available to the two unit coordinators (DG-R and BH) at the end of a study period.

#### Retention

Administrative data was available on the number of students who enrolled in the unit, and of these the number who withdrew, failed and passed the unit. The retention rate was calculated as the sum of the number of students who passed or failed the unit divided by total enrolments.

### Procedure and Unit Timing

Prior to the research commencing approval was obtained from Curtin University’s Human Research Ethics Committee (HREC approval number: PSYCH & SP 1013–68). As only de-identified administrative data was used in the study, consent from students was not required. Existing data files on student grades (already held by the unit coordinators DG-R and BH) were de-identified in preparation for analysis. Aggregated de-identified student evaluation data and retention summaries for the unit were downloaded from university systems.

Students completed the online and face-to-face version of the unit over the same timeframe (Semester 1, 2013), with identical assessment due dates. The first assignment, which was the written assignment, was due in the 4th week of semester. The electronic test was in 8th week (7th teaching week) of semester, and the examination was in the examination period following semester completion. The face-to-face students completed their group presentation assignment in weeks 11 or 12, in front of their tutorial class. For the online students, the group presentation was submitted online before the end of week 12. All assessments in both versions of the unit were marked by the same marking team, consisting of the two unit coordinators and the face-to-face tutors.

### Statistical Analysis

Independent samples *t*-tests were conducted to test differences in student marks by mode of delivery. Bootstrapping procedures employing 10000 bootstrapped samples were used to reduce the impact of univariate non-normality (Field, 2013), with confidence intervals (*CIs*) reported as bias corrected and accelerated (*BcA*) intervals for purposes of analysis rigor. Chi square analyses were used to compare retention rates by mode of delivery. Effect sizes for all analyses were interpreted using Cohen’s (1992) effect size conventions.

## Results

### Quantitative Results

Descriptive statistics of student marks by mode of delivery are provided in **Table 2**. Students in the face-to-face unit (*M* = 73.25, *SD* = 9.04) had significantly higher final marks compared to students in the online unit (*M* = 68.96, *SD* = 11.17), *t*(44.05) = 2.45, *p* = 0.018, *95% BcA CI* (1.12, 7.40). The estimate of effect size, *d* = 0.47, was representative of a small to medium effect size. A higher percentage of face-to-face students (93%) passed the unit compared to online students (80%), with 11% of online students failing (9% withdrew) compared to 4% of face-to-face students (4% withdrew).

**Table 2:** Semester weighted average and assessment task marks (mean and standard deviation) by mode of delivery (face-to-face/online).

	Face-to-face (*N* = 752)	Online (*N* = 42)
Semester weighted average	73.25 (9.04)	68.96 (11.17)
Written assignment	12.91 (2.91)	11.87 (3.75)
Electronic test	21.28 (3.94)	21.38 (4.42)
Presentation	24.43 (2.84)	21.49 (3.26)
Examination	32.63 (6.15)	32.02 (7.41)

*Participants include all students who completed all assessments, whether or not they passed the unit.*

Given the finding of a significant difference in final marks, further independent samples *t*-tests were conducted to determine the assessment components on which students in the face-to-face and online units differed. There were no significant differences in scores between the two units on the written assignment, electronic test or examination. However, students in the face-to-face unit (*M* = 24.43, *SD* = 2.84) scored significantly higher than students in the online unit (*M* = 21.49, *SD* = 3.26) on the group presentation assessment, *t*(792) = 6.49, *p* < 0.001, *95*% *BcA CI* (2.05, 3.83).

The percentage of students agreeing with each item on the student evaluation questionnaire are presented by study mode in **Table 3**. For each item, more than 80% of students responding indicated that they were satisfied^1^. For all but two items, the percentage of students indicating satisfaction in both face-to-face and online units was higher than the university average, indicating high satisfaction with the unit. In the face-to-face unit 97% (with 2% unable to judge) of students indicated that they were satisfied with the unit overall, compared to 89% (with 6% unable to judge) of students in the online unit.

**Table 3:** Student evaluation ratings (percentage agreement) for each item by mode of delivery for the introductory psychology unit with comparison to university average.

Item	Face-to-face (*N* = 388)	Online (*N* = 23)	University
1. The learning outcomes in this unit are clearly identified.	97	96	89
2. The learning experiences in this unit help me to achieve the learning outcomes.	96	96	85
3. The learning resources in this unit help me to achieve the learning outcomes.	95	91	85
4. The assessment tasks in this unit evaluate my achievement of the learning outcomes.	95	87	85
5. Feedback on my work in this unit helps me to achieve the learning outcomes.	90	91	79
6. The workload in this unit is appropriate to the achievement of the learning outcomes.	98	100	86
7. The quality of teaching in this unit helps me to achieve the learning outcomes.	93	100	84
8. I am motivated to achieve the learning outcomes in this unit.	96	87	86
9. I make best use of the learning experiences in this unit.	92	83	87
10. I think about how I can learn more effectively in this unit.	97	83	86
11. Overall, I am satisfied with this unit.	98	91	84

We also compared retention rates of students in the face-to-face unit snd online unit. **Table 4** presents a breakdown of the number of students enrolled in the face-to-face and online units by whether they withdrew, passed or failed the unit. The retention rate (students who passed or failed/total number of students) was significantly higher for face-to-face unit students (96%) than online unit students (91%); χ^2^ (1, *N* = 866, *p* = 0.046). Withdrawal and failure rates were more than twice as high in the online unit.

**Table 4:** Completion status of students by mode of delivery (face-to-face/online).

	Face-to-face		Online	
Status	*N*	%	*N*	%
Passed	752	93%	45	80%
Failed	29	4%	6	11%
Withdrawn	29	4%	5	9%
Total	810		56	

### Qualitative Results

A content analysis was conducted of comments made by online students on their unit evaluation surveys. Seventeen online students commented on the most helpful aspect of the unit and 13 online students commented on how the unit could be improved. LR and DG-R agreed on the summary of the content analysis.

Ten students commented positively on the structure of the unit, noting that the lecture segments interspersed with activities aided learning. For example:

“The way the module was structured was very helpful, I really liked the pace of the unit. I found that the shorter lecture videos, divided up between reading content and external videos, made the learning varied and different to other units. It made my experience enjoyable and I believe that it helped me understand the content better than if the unit was structured with the standard lecture and readings that other units are based on.”

Eight students commented positively on the lecture segments specifically, enjoying the variety of topics and skilled presenters. For example:

*“The material is very well presented by very competent lecturers who focus on articulating key ideas”*.

The inclusion of video of the presenters was also perceived as beneficial to student engagement. For example:

*“…made the overall experience more engaging and personal, which is a huge hurdle to overcome when your are a* [sic] *external student”*

“[lecturer] *overflows with enthusiasm for his topics and this means that his lectures are presented in a very entertaining and engaging style”*.

However, one student did not like the use of short lecture segments, commenting that they

“prefer 2 h long for ease of downloading and storing.”

Seven students commented positively on interactions with the teaching staff, in particular noting the prompt feedback to questions. Students commented on the rapidity of responding to student queries, for example:

*“The lecturers always responding quickly with answers to any questions posted on the discussion board”*.

Only one student commented on the limited opportunities for engagement with staff: “*One aspect I struggled with as an external was the lack of contact time with tutors. And for all it’s worth the blogs and emails only really scape* [sic] *the surface. It is when there is discussion that is live and instant that progress is made*”.

The most common area identified for improvement was the group assignment, with six students commenting on this, representing 46% of the comments related to unit improvements. Social loafing was voiced as a problematic facet of the online group assessment task. As one student commented:

*“My experience this time with the group assignment was fine, but it easily could have been a very difficult situation to achieve a high mark in if I had been lumbered with two social loafers rather than 1! I personally would prefer not to do group assignments in this way. I have worked very hard to achieve a high result in this unit and the group assignment could easily have substantially reduced my overall mark through no fault of my own, and I did over and above my share of the work to ensure my grade would not be compromised*”.

No other areas for improvement were suggested by more than one student. To investigate if the group work assignment was a particular source of dissatisfaction for online students only, a search was made of the student comments on how the face-to-face version of the unit could be improved. Only 10 comments (4.8% of comments) related to the group assignment, with most of these focussing on process issues and/or the need for clearer instructions.

## Discussion

The current study aimed to compare student grades, student satisfaction and retention rates in online and face-to-face versions of an introductory psychology unit, where the online version had been developed to provide equivalent learning experiences to the face-to-face version.

On average, overall student grades were lower for online students than students in the face-to-face unit. However, this overall effect masked differences by assignment. Only marks for the group presentation assignment were significantly lower for online students than students in the face-to-face version of the unit. This is particularly interesting as the group work assignment was the only assignment that had variation in format and marking criteria between the two versions of the unit, in order to accommodate geographical and technological limitations. Specifically, students in the online version of the unit were not required to orally present their work, while this was a requirement for students in the face-to-face unit. These findings could be taken to suggest that face-to-face and online students will perform at an equivalent level in equivalent assessments (such as the examination, electronic test, and written assignment here) but that differences in assignment structure and/or marking criteria (as per the group work assignment) may produce non-equivalent outcomes. An implication of this finding is that when units are offered in multiple delivery modes, assessments should ideally be identical in all modes (i.e., student requirements, marking criteria, etc.) in order to promote equivalent learning outcomes. This is consistent with Equivalency Theory (Simonson, 1999) and with the assertions of previous researchers (e.g., Clark, 1994) that the manner of material delivery, rather than the format through which it is delivered, is the key variable in terms of student learning and outcome equivalency. Because students were not randomly allocated to study mode and that, consequently, we cannot discount the possibility of pre-existing group differences in academic ability that might be masked by the current findings.

Our findings indicated high rates of satisfaction for both online and face-to-face students. One probable reason for the high levels of satisfaction expressed by online students is that the online unit, in an attempt to ensure equivalence to the face-to-face unit, utilized a multimedia approach to the delivery of materials, including lecture material. Boling et al. (2012) found that online students preferred online courses using multimedia and high levels of interaction over those which were based more strongly on text-based resources. In addition, qualitative comments generally indicated that students were satisfied with the accessibility of, and timely feedback from, instructors. This is consistent with previous research in this area (e.g., Reisetter et al., 2007; Boling et al., 2012), and the instructors’ ability to provide this interaction and feedback was enhanced because the online unit was similar in structure and timing to the face-to-face unit that was running concurrently. Hence, designing online units to be equivalent to face-to-face units may enhance ease of instruction, benefitting students.

Analysis of student comments in the online unit identified group work as the main area of dissatisfaction. It is possible that this is, to some extent, to be expected in online units given evidence that online students tend to be more independent, self-directed learners (Leasure et al., 2000; Meyer, 2003) and consequently might prefer to work independently. Vance et al. (2015) compared online and face-to-face students’ attitudes toward group work, reporting that online students had more negative attitudes toward teamwork than face-to-face students. Previous research has identified a range of other factors that are associated with dissatisfaction with group work in online units. Tseng and Yeh (2013) identified lack of communication, low levels of individual accountability and questionable behaviors by other group members as negatively affecting teamwork online. Boling et al. (2012) found that group work did not improve sense of community in an online unit. Consequently, our findings and those outlined prior suggest that collaborative group-based assessments may present particular difficulties when attempting to design course assessments for equivalency between modes of delivery. While experimental research with students randomly assigned to online or oﬄine conditions would clarify whether differences in grades and satisfaction with group work are attributable to the mode of delivery rather than self-selection, the reality is that students will continue to self-select into courses. As such, while our study has low internal validity, it has high ecological and external validity.

We also note that low satisfaction with group work is not restricted to online courses. For example, almost a third of a sample of Australian university students responding to a survey on group work indicated that they had endured poor or very poor group work experiences, with key sources of dissatisfaction relating to group marks not reflecting individual contributions, differing work styles, group formation, and division of tasks (Hall and Buzwell, 2012). Nevertheless, given the current finding that the only consistent source of dissatisfaction with an online introductory psychology unit was a group work assessment, it appears pertinent for future research to investigate methods to increase online student satisfaction and performance in this type of assessment. This is particularly important in psychology units, where meta-analytic findings have indicated strong positive learning as a result of collaborative learning (via small-group) activities (Tomcho and Foels, 2012). Group work is also a requirement in undergraduate psychology programs due to course accreditation requirements, stipulating that graduates must be able to “Demonstrate effective oral communication skills in various formats (e.g., debate, group discussion, presentation) and for various purposes.” and … “collaborate effectively, demonstrating an ability to: work with groups to complete projects within reasonable timeframes; manage conflicts appropriately and ethically” (Cranney et al., 2009, p. 259). Further research is required on how best to facilitate learning through group work in both online and traditional psychology units.

The current study found significantly higher rates of retention for face-to-face compared to online students. This finding should be interpreted with some caution, given the quasi-experimental methodology, the small sample size of online students. and relatively small difference in retention percentages. However, the findings are consistent with previous research (e.g., Maki et al., 2000; Neff and Donaldson, 2013). It has been suggested that withdrawal from online units is likely to be related more to the characteristics of students who tend to enroll in online courses, rather than characteristics of the courses themselves (Rodriguez, 2011). For example, qualitative research with expert online faculty indicated the importance of student self-discipline in relation to retention (Gaytan, 2015). Quantitative analysis has identified relevant student characteristics predicting retention are academic experience, previous academic performance and previous withdrawal from online units (Cochran et al., 2014). Assuming that this difference in retention remains in studies utilizing larger samples of online students, future research could investigate pedagogical and course factors related to student withdrawal from online units in an effort to enhance student retention, and promote successful learning. A limitation of our findings on online unit retention is that the high levels of satisfaction reported in the current online unit are only relevant to students who completed the unit, and it is possible that students who withdrew were dissatisfied with other aspects of online study. Future research could investigate this prospect further through targeted research with students who withdraw from online courses. Research to date has focused on interviewing enrolled students about retention (e.g., Gaytan, 2015), rather than students who have already withdrawn, reflecting limited knowledge about *actual* student retention with regard to online unit enrolment.

In summary, we have presented a quasi-experimental study that compares student grades, student satisfaction and retention rates in online and face-to-face versions of an introductory psychology unit designed to provide equivalent learning experiences. Although results must be interpreted cautiously because of the quasi-experimental nature of the research and the relatively small online sample (limiting generalisability), they support Equivalency Theory’s (Simonson, 1999) proposition that online and classroom-based learners will attain equivalent learning outcomes when equivalent learning experiences are afforded. These findings additionally contribute to the body of evidence required to determine the effectiveness of online psychology units (Halonen et al., 2013). In this study, the one learning activity/assessment that appears not to have provided equivalent learning experiences, the group presentation assessment, was associated with differences in marks, and accompanied by student dissatisfaction. Further research is required to determine effective methods of engaging students in online group activities. Future research using validated measures of student satisfaction will enable a more nuanced analysis of student satisfaction that was not possible with the aggregated student satisfaction data used in this study. Consistent with previous research, retention rates were lower in the online unit, indicating the need for further research to develop effective strategies to increase online retention rates.

## Author Contributions

DG-R was responsible for the conceptualization and theoretical foundations of the research. He was primarily responsible for writing and editing the manuscript. LR was primarily responsible for the qualitative data analysis and was also involved in the editing of the manuscript. BH was responsible for the quantitative data analysis and contributed to final manuscript editing.

## Conflict of Interest Statement

The authors declare that the research was conducted in the absence of any commercial or financial relationships that could be construed as a potential conflict of interest.

## Footnotes

1 The university target is 80% of students agreeing with each item.
